# Proteomic analyses in diverse populations improved risk prediction and identified new drug targets for type 2 diabetes

**DOI:** 10.2337/dc23-2145

**Published:** 2024-04-16

**Authors:** Pang Yao, Andri Iona, Alfred Pozarickij, Saredo Said, Neil Wright, Kuang Lin, Iona Millwood, Hannah Fry, Christiana Kartsonaki, Mohsen Mazidi, Yiping Chen, Fiona Bragg, Bowen Liu, Ling Yang, Junxi Liu, Daniel Avery, Dan Schmidt, Dianjianyi Sun, Pei Pei, Jun Lv, Canqing Yu, Michael Hill, Derrick Bennett, Robin Walters, Liming Li, Robert Clarke, Huaidong Du, Zhengming Chen, on behalf of China Kadoorie Biobank Collaborative Group, International Steering Committee, Junshi Chen, Zhengming Chen, Robert Clarke, Rory Collins, Liming Li, Chen Wang, Jun Lv, Richard Peto, Robin Walters, International Co-ordinating Centre, Oxford, Daniel Avery, Derrick Bennett, Ruth Boxall, Sushila Burgess, Ka Hung Chan, Yiping Chen, Zhengming Chen, Johnathan Clarke, Robert Clarke, Huaidong Du, Ahmed Edris Mohamed, Hannah Fry, Simon Gilbert, Pek Kei Im, Andri Iona, Maria Kakkoura, Christiana Kartsonaki, Hubert Lam, Kuang Lin, James Liu, Mohsen Mazidi, Iona Millwood, Sam Morris, Qunhua Nie, Alfred Pozarickij, Paul Ryder, Saredo Said, Dan Schmidt, Becky Stevens, Iain Turnbull, Robin Walters, Baihan Wang, Lin Wang, Neil Wright, Ling Yang, Xiaoming Yang, Pang Yao, National Co-ordinating Centre, Beijing, Xiao Han, Can Hou, Qingmei Xia, Chao Liu, Jun Lv, Pei Pei, Dianjanyi Sun, Canqing Yu, 10 Regional Co-ordinating Centres, Guangxi Provincial CDC, Naying Chen, Duo Liu, Zhenzhu Tang, Liuzhou CDC, Ningyu Chen, Qilian Jiang, Jian Lan, Mingqiang Li, Yun Liu, Fanwen Meng, Jinhuai Meng, Rong Pan, Yulu Qin, Ping Wang, Sisi Wang, Liuping Wei, Liyuan Zhou, Gansu Provincial CDC, Caixia Dong, Pengfei Ge, Xiaolan Ren, Maiji CDC, Zhongxiao Li, Enke Mao, Tao Wang, Hui Zhang, Xi Zhang, Hainan Provincial CDC, Jinyan Chen, Ximin Hu, Xiaohuan Wang, Meilan CDC, Zhendong Guo, Huimei Li, Yilei Li, Min Weng, Shukuan Wu, Heilongjiang Provincial CDC, Shichun Yan, Mingyuan Zou, Xue Zhou, Nangang CDC, Ziyan Guo, Quan Kang, Yanjie Li, Bo Yu, Qinai Xu, Henan Provincial CDC, Liang Chang, Lei Fan, Shixian Feng, Ding Zhang, Gang Zhou, Huixian CDC, Yulian Gao, Tianyou He, Pan He, Chen Hu, Huarong Sun, Xukui Zhang, Hunan Provincial CDC, Biyun Chen, Zhongxi Fu, Yuelong Huang, Huilin Liu, Qiaohua Xu, Li Yin, Liuyang CDC, Huajun Long, Xin Xu, Hao Zhang, Libo Zhang, Jiangsu Provincial CDC, Jian Su, Ran Tao, Ming Wu, Jie Yang, Jinyi Zhou, Yonglin Zhou, Suzhou CDC, Yihe Hu, Yujie Hua, Jianrong Jin, Fang Liu, Jingchao Liu, Yan Lu, Liangcai Ma, Aiyu Tang, Jun Zhang, Qingdao CDC, Liang Cheng, Ranran Du, Ruqin Gao, Feifei Li, Shanpeng Li, Yongmei Liu, Feng Ning, Zengchang Pang, Xiaohui Sun, Xiaocao Tian, Shaojie Wang, Yaoming Zhai, Hua Zhang, Licang CDC, Wei Hou, Silu Lv, Junzheng Wang, Sichuan Provincial CDC, Xiaofang Chen, Xianping Wu, Ningmei Zhang, Weiwei Zhou, Pengzhou CDC, Xiaofang Chen, Jianguo Li, Jiaqiu Liu, Guojin Luo, Qiang Sun, Xunfu Zhong, Zhejiang Provincial CDC, Weiwei Gong, Ruying Hu, Hao Wang, Meng Wang, Min Yu, Tongxiang CDC, Lingli Chen, Qijun Gu, Dongxia Pan, Chunmei Wang, Kaixu Xie, Xiaoyi Zhang

**Affiliations:** 1 Clinical Trial Service Unit & Epidemiological Studies Unit, Nuffield Department of Population Health, University of Oxford, Oxford, UK; 2 Medical Research Council Health Research Unit, Nuffield Department of Population Health, University of Oxford, Oxford, UK; 3 Department of Epidemiology and Biostatistics, School of Public Health, Peking University Health Science Center, Beijing, China; 4 Peking University Center for Public Health and Epidemic Preparedness & Response, Beijing, China; 5 Key Laboratory of Epidemiology of Major Diseases (Peking University), Ministry of Education, Beijing, China

**Keywords:** Proteomics, Prospective studies, T2D, Genetics, Risk prediction, Drug targets

## Abstract

**Objective:**

Integrated analyses of plasma proteomics and genetic data in prospective studies can help assess the causal relevance of proteins, improve risk prediction and discover novel protein drug targets for T2D.

**Research Design and Methods:**

We measured plasma levels of 2923 proteins using OLINK Explore among ~2000 randomly selected participants from CKB without prior diabetes at baseline. Cox regression assessed associations of individual protein with incident T2D (n=92 cases). Proteomic-based risk models were developed with discrimination, calibration, reclassification assessed using AUC, calibration plots and NRI, respectively. Two-sample MR analyses using *cis*-pQTLs identified in GWAS of CKB and UKB for specific proteins were conducted to assess their causal relevance for T2D, along with colocalization analyses to examine shared causal variants between proteins and T2D.

**Results:**

Overall 33 proteins were significantly associated (FDR<0.05) with risk of incident T2D, including IGFBP1, GHR and amylase. The addition of these 33 proteins to conventional risk prediction model improved AUC from 0.77 (0.73-0.82) to 0.88 (0.85-0.91) and NRI by 38%, with predicted risks well calibrated with observed risks. MR analyses provided support for the causal relevance for T2D of ENTR1, LPL and PON3, with replication of ENTR1 and LPL in Europeans using different genetic instruments. Moreover, colocalization analyses showed strong evidence (PH4>0.6) of shared genetic variants of LPL and PON3 with T2D.

**Conclusion:**

Proteomic analyses in Chinese adults identified novel associations of multiple proteins with T2D with strong genetic evidence supporting their causal relevance and potential as novel drug targets for prevention and treatment of T2D.

## Graphical abstract

**Figure:**
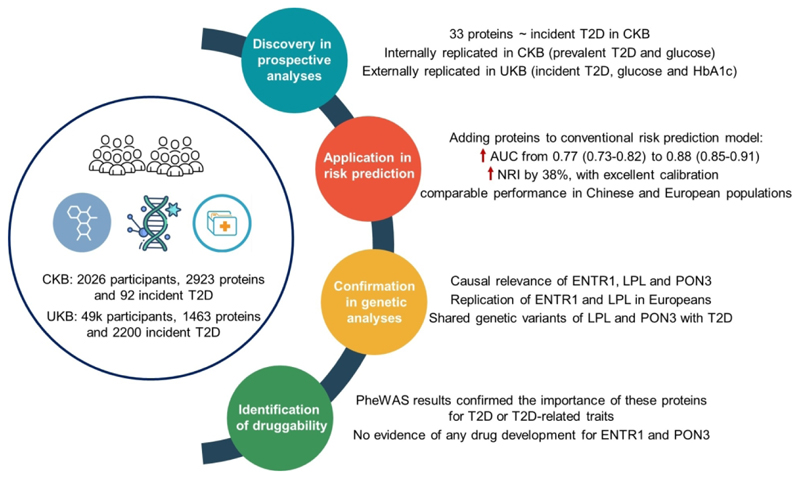

## Introduction

Globally type 2 diabetes (T2D) affects >530 million adults,^1^ causing substantial risks of premature death and macro- and micro-vascular complications. China has the largest number of people with diabetes (>140 million) in the world and the prevalence is still rising.^1^ Several important modifiable risk factors for T2D are established (e.g. adiposity, lack of physical activity and suboptimal diet), which account for about 70% of new cases globally.^2^ These risk factors have been widely used, typically in combination with blood glucose and/or HbA1c, to predict risk of T2D and inform prevention and treatment decision in diverse populations.^3, 4^ Recently, GWAS of T2D in diverse populations identified >240 common genetic variants, including >180 in East Asian populations.^5, 6^ However, the mechanisms underlying many of these associations remain to be elucidated. Plasma proteins play a central role in human biology and represent a primary source of therapeutic targets.^7^ Analyses of circulating protein biomarkers, particularly when integrated with genetic data, in population and clinical studies, can help clarify disease aetiology, improve risk prediction and early diagnosis, and discover novel and repurposing therapeutic targets for treatment of T2D and other major diseases.^8–12^

Previous studies of plasma proteins and T2D have highlighted the roles of several specific proteins (e.g. IGFBP1, IGFBP2, GHR and SHBG) in aetiology of T2D.^12–16^ Advances in high throughput proteomic assays now enable measurement of several thousand proteins,^17–19^ and their application in population and clinical studies of primarily European ancestry populations have identified several novel protein biomarkers for T2D.^12–16^ However, little is known about the relevance of protein biomarkers for T2D in non-European ancestry populations including Chinese where the disease rates, distribution of risk factors and genetic architecture differ greatly from European ancestry populations. Moreover, few previous studies undertook detailed genetic analyses (e.g. MR and colocalization analyses) to assess the causal relevance of specific proteins for T2D.^16, 20^

We undertook an integrated analysis of observational and genetic data of ~3000 proteins with incident T2D in ~2000 adults selected from the China Kadoorie Biobank. The present report aims to: (i) identify plasma proteins significantly associated with incident T2D; (ii) assess the utility of selected proteins for prediction of T2D risk; (iii) use *cis*-pQTLs identified in GWAS for proteins to assess their causal relevance for T2D via two-sample MR and separate colocalization analyses; and (iv) clarify the mechanisms of action for specific proteins using PheWAS, tissue expression and other experimental evidence.

## Methods

### Study population and data collection

Details of the CKB study design, methods, and participants have been previously reported.^21^ Briefly, a total of 512,715 participants aged 30-79 years were enrolled from 10 (5 urban, 5 rural) geographically diverse regions in China. At baseline (June 2004-July 2008) and at three subsequent resurveys in a ~5% subset (in 2008, 2014 and 2021, respectively), detailed data were collected on socio-demographic characteristics, smoking, alcohol consumption, diet, physical activity, personal (e.g. IHD, stroke and T2D) and family medical history, along with physical and blood measurements (e.g. blood pressure, BMI, and random blood glucose but not HbA1c).

Follow-up of CKB participants was through linkage to established mortality (cause-specific) and morbidity (including T2D) registries, and to the nationwide health insurance system which records all hospitalised episodes.^21^ All disease events and causes of death were ICD-10 coded by trained health workers, blinded to baseline information, and checked and integrated centrally. Ethical approval was obtained from relevant international, national and regional ethics committees or institutional research boards.^21^ All participants provided written informed consent.

The present study involved 2026 participants selected as a subcohort for a nested case-subcohort study of IHD in CKB.^22^ They were randomly selected from a population subset of 69,353 genotyped participants who had no prior history of CVD nor statin use at baseline and were genetically unrelated to each other. Among these 2026 participants, 130 having prevalent diabetes at baseline who were analysed separately for internal replication but excluded from the main analyses. During 11 years of follow-up (up to 1.1.2018), 92 individuals developed incident T2D (ICD10: E10-E14) among the 1896 participants included in the main analyses.

### Proteomics assay

Stored baseline plasma samples from participants were retrieved, thawed, and sub-aliquoted to multiple aliquots, with one aliquot (100 μL; for batch 1 assay) shipped on dry ice to the OLINK Biosciences Laboratory at Uppsala, Sweden, and one aliquot (for batch 2 assay) shipped subsequently to OLINK lab at Boston, USA, for multiplex proximity extension assay of proteins. Batch 1 covered 1463 unique proteins first released by the OLINK, while the batch 2 covered a further 1460 unique proteins released subsequently by the OLINK. To minimize inter- and intra-run variation, the samples were randomized across plates and normalized using both an internal control (extension control) and an inter-plate control and then transformed using a pre-determined correction factor.

Details of the OLINK assay performance and validation have been previously reported.^18^ The LOD were determined using negative control samples (buffer without antigen). A sample is flagged as having QC warning if incubation control deviates more than a pre-determined value (±0.3) from the median value of all samples on the plate. The pre-processed data were provided in the arbitrary unit Normalized Protein eXpression (NPX) on a log2 scale. The present analyses has a total of 2941 proteins (2923 unique proteins), including 1472 proteins (1463 unique proteins) in batch 1 and 1469 proteins (1460 unique proteins) in batch 2.

### Statistical analysis

Plasma protein levels were standardized (i.e. values of each protein were divided by their SD) and analysed as continuous variables. In observational analysis, Cox and logistic regression models were used to estimate adjusted HRs and ORs (and 95% CI) for incident and prevalent diabetes, respectively. All analyses were adjusted for age, age^2^, sex, study area, fasting time, ambient temperature, plate ID, education (4 categories: no formal school, primary school, secondary school, high school and above), smoking (3 categories: never, occasional or ex-regular, and regular smoker), alcohol drinking (3 categories: never, occasional or ex-regular, and weekly), physical activity (MET-h), family history of diabetes (binary) and BMI. Sensitivity analyses were conducted by further adjusting dietary variables, and by adjusting the effect of plate using residual from regression of protein values on plate as a covariate in the models. The cross-sectional analysis for random plasma glucose (per SD) was restricted to participants without prior diabetes at baseline. Analyses were also conducted in UKB for diabetes incidence, blood glucose and HbA1c (see eAppendix).

For proteins significantly associated with incident diabetes, we further (i) examined the shape of the associations with T2D by quartiles of individual proteins; (ii) assessed the performance of proteomic-based risk prediction of T2D in CKB, with discrimination and calibration utilities assessed using AUC and calibration plots with the Hosmer–Lemeshow test, respectively. Reclassification was measured using both the percentile-based NRI with deciles of relative risk as reference categories, and the continuous NRI. The proteomic risk model was internally validated using 1000 bootstrap method and compared and combined with conventional risk prediction model in Chinese,^23^ with further external validation in the UKB; and iii) conducted GO and KEGG enrichment analyses using clusterProfiler (v.4.2.2),^24^ to determine which biological functions or processes were significantly enriched based on hypergeometric tests.

For proteins showing significant associations with T2D in observational analyses, a two-sample MR using Wald ratio method,^25, 26^ was conducted using (i) *cis*-pQTLs obtained from GWAS of CKB, with lookups in AGEN consortium of East Asian adults including 66,677 diabetes cases;^6^ and (ii) *cis*-pQTLs obtained from GWAS of UKB, with lookups in the DIAMANTE Consortium of European descent including 80,154 diabetes cases and 853,816 controls.^5^ Colocalization was performed only for those proteins that had 95% credible sets identified by fine mapping in both AGEN T2D and CKB *cis*-pQTL datasets. Fine mapping was performed using susieR (v0.12.16) and colocalization was performed using coloc (v5.2.1) packages in R.

For proteins showing significant genetic associations with T2D, we screened protein expression database of GTEx to study the tissue-specific role of the causal proteins in diabetes. We further searched T2DKP for associations of (i)) *cis*-pQTLs from both CKB and UKB with a range of phenotypes using a *P* value threshold of 5×10^-8^ and (ii) genes with available diseases and traits using a HuGE Scores threshold of 10, indicating stong evidence for the causal relevance of such proteins for diseases or traits.^27^

**Figure 1:**
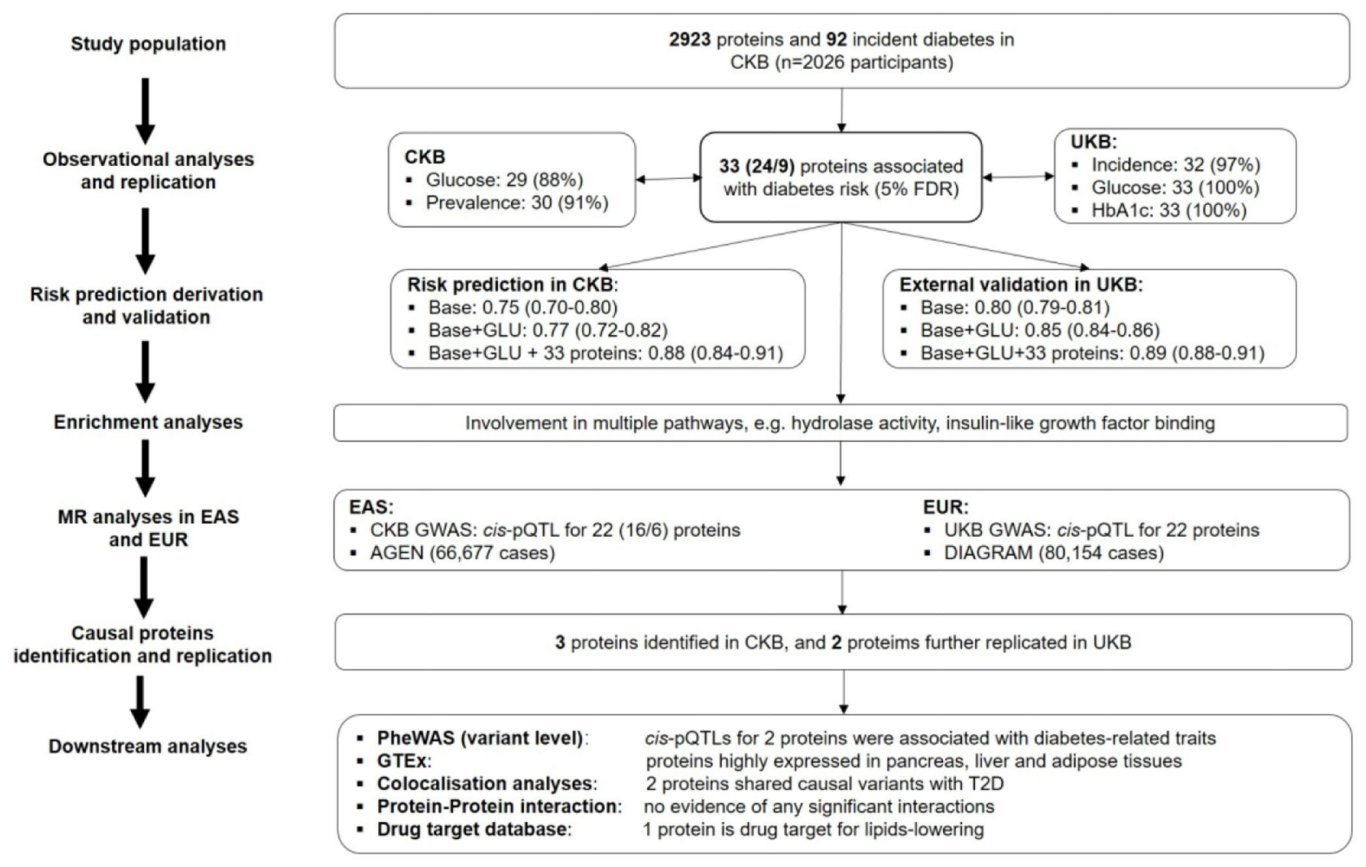
Overview of study design, analytic approaches and key findings

Figure 1 provides an overview of main analytic approaches. All statistical analyses were performed using R version 4.1.2. Benjamini-Hochberg FDR was used to correct for multiple testing.

## Results

Among the 1896 participants included in the main analyses, the mean (SD) age was 51.3 (10.4) years, 62.1% were women, and 50.6% were urban residents, which, along with many other baseline characteristics, were similar to those in the overall genotyped CKB cohort (eTable 1).

### Observational associations of proteins with diabetes

**Figure 2:**
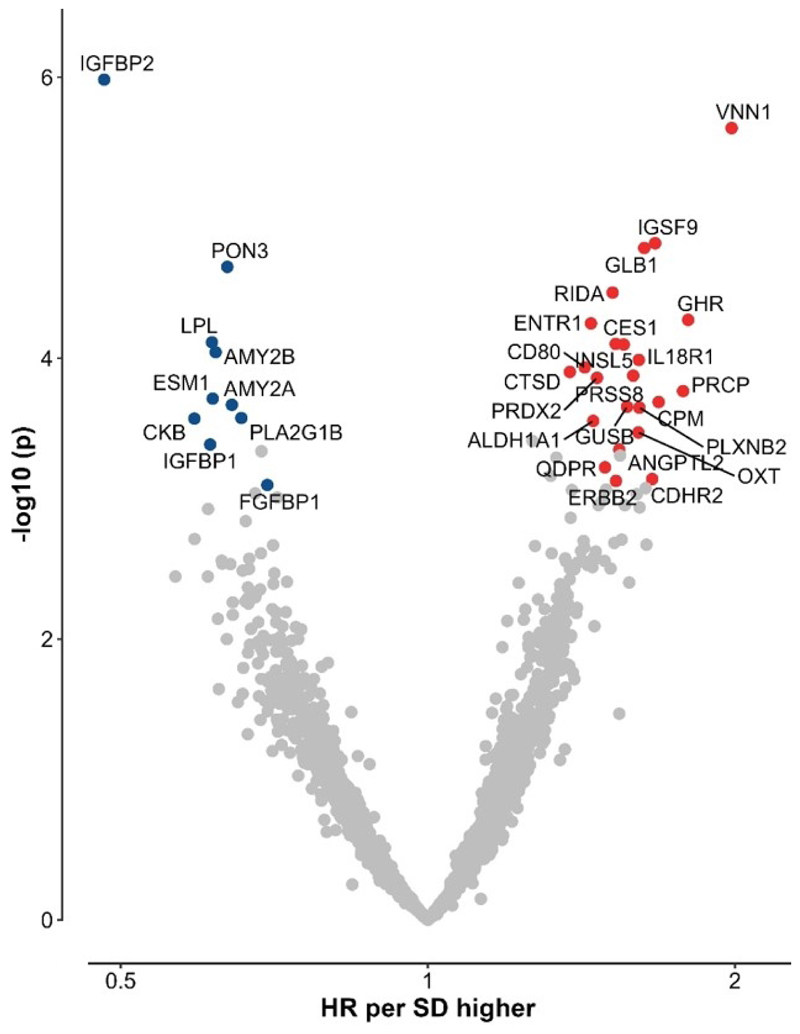
Associations of 1-SD higher levels of 2941 proteins with incident diabetes in observational analyses Models were adjusted for age, age^2^, sex, study area, fasting time, ambient temperature, plate ID, education, smoking, alcohol consumption, physical activity, family history of diabetes and BMI. Red, blue and grey dots denote significant positive, significant inverse and non-significant associations, respectively.

After adjusting for conventional risk factors, 33 proteins were significantly associated at 5% FDR with risk of T2D (batch 1/2: 24/9) (Figure 2).The associations were typically log-linear throughout the full ranges of levels of specific proteins examined (eFigure 1), although the adjusted HRs (per 1SD higher protein level) varied, from 1.38 to 1.98 for those showing positive associations (23 proteins) and from 0.48 to 0.70 for those showing inverse associations (10 proteins) with T2D (eFigure 2). Proteins showing the strongest positive associations were VNN1 (1.98, 95% CI 1.49-2.64), GHR (1.80, 1.35-2.39), PRCP (1.78, 1.32-2.40), CPM (1.68, 1.28-2.22) and IGSF9 (1.67, 1.32-2.11). The proteins showing strongest inverse associations included IGFBP2 (0.48, 0.36-0.65), CKB (0.59, 0.44-0.78), IGFBP1 (0.61, 0.47-0.80), LPL (0.61, 0.48-0.78) and ESM1 (0.62, 0.48-0.69). Six proteins (IGFBP2, VNN1, IGSF9, GLB1, PON3, and RIDA) were significantly associated with incident T2D after applying Bonferroni multiple test correction. Further adjustments for fresh fruit, red meat consumption and a healthy diet score did not alter the results, as were use of the residual from regression of protein values on plate as a covariate in the models.

In internal replication analyses, most of these 33 proteins were significantly associated with blood glucose (29/33; 88%) levels or prevalent diabetes (30/33; 91%) (eFigure 3). Moreover, all 33 proteins were externally replicated in UKB (at 5% FDR) for blood glucose, HbA1c, and incident T2D (except one protein), although the effect sizes varied (eFigure 4). Of these 33 proteins, most proteins had similar HRs in observational analyses of both studies, while the HRs for 3 proteins (VNN1, GHR, IGFBP2) differed somewhat but all directionally concordant with each other.

These 33 proteins were only moderately correlated with each other, with 99.5% of protein pairs having correlation coefficients ranging from -0.7 to +0.7 (eFigure 5).

### Risk prediction of incident T2D

In CKB the conventional risk prediction model without blood glucose had an AUC of 0.754 (0.710-0.798), increasing to 0.774 (0.730-0.818) with addition of blood glucose (Table 1). Proteomic-based model (33 proteins) alone had AUC of 0.824 (0.786-0.862), which significantly outperformed the conventional models. The addition of top 10 or all 33 proteins to conventional risk factors plus blood glucose model yielded an AUC of 0.844 (0.803-0.885) and 0.876 (0.846-0.906), respectively. For NRI the corresponding values were 28% (15-41%) and 38% (24-52%), respectively, using categorical approach, rising to 84% (65%-103%) and 97% (79%-115%) when using continuous approach (eTable 2). The observed and predicted risk of T2D showed excellent calibration (the Hosmer–Lemeshow test: χ^2^=3.4, *P*=0.90; eFigure 6). The application of these same proteins identified in CKB to UKB yielded comparable results for prediction of T2D (eTables 3-4).

### Enrichment analysis

In enrichment analyses of 33 proteins, hydrolase activity was identified as the top biological pathway (eFigure 7). Other pathways such as growth factor binding and insulin-like growth factor binding, were also among the top overrepresented biological pathways. None of the pathways were annotated after correction for multiple testing in similar analyses using KEGG method.

### Genetic associations

In CKB GWAS, *cis*-pQTL variants were identified for 22 (67%) of the 33 proteins. In two-sample MR analyses involving CKB and AGEN that excluded CKB, 3 proteins (ENTR1, LPL, PON3) were significantly associated at FDR<0.05 with T2D (Table 2). Of these 3 proteins, the HRs were less extreme in MR than in observational analyses but were directionally concordant. Moreover, colocalization analyses provided strong support (PH4>0.6) for shared genetic variants of two proteins (LPL and PON3) with T2D. Independent two-sample MR analyses involving 22 *cis*-pQTLs identified in UKB GWAS for these 33 T2D-associated proteins replicated associations for ENTR1 (*P*=0.004) and LPL (*P*=0.01). For PON3, although *cis*-pQTL was identified in UKB GWAS the association (with same direction) was not significant (*P*=0.49).

### PheWAS and drug target lookup

In PheWAS analyses of these 3 proteins, *cis*-pQTL for ENTR1 were associated with T2D and several T2D-related traits, including HbA1c, insulin and glucose (Table 3). Likewise, *cis*-pQTLs for LPL and PON3 were related to T2D, CVD outcomes (MI and CAD) and CVD risk factors (LDL, TG, ApoA). Within CKB, ENTR1, LPL and PON3 were each significantly associated with glucose in cross-sectional analyses. All three proteins were highly expressed in liver, pancreas and adipose tissues and additional analyses of single-gene KO mouse models identified associations with several lipidaemia-related phenotypes (LPL, ENTR1 and PON3), abnormal liver morphology (ENTR1) and oxidative stress (PON3). Analysis of Open Targets and other databases indicated evidence of drug development for one protein (LPL), including commercially available drug of Ibrolipim, a lipoprotein lipase activator that degrades circulating triglycerides in blood (Table 2). However, there were no reports of drug targets or development for ENTR1 and PON3.

## Discussion

In this study of Chinese adults, we found 33 proteins were significantly associated with risk of incident T2D. The addition of these proteins to the conventional prediction models substantially improved risk prediction of T2D, with comparable performance in Chinese and European populations. Moreover, MR analyses based on *cis*-pQTLs identified in CKB GWAS provided strong support for the causal relevance of three proteins (ENTR1, LPL and PON3) for T2D, with replication of ENTR1 and LPL in Europeans using different *cis*-pQTLs. In colocalization analyses, there was strong evidence of shared causal genetic variants of T2D with two proteins (LPL and PON3). Furthermore, the PheWAS results confirmed the importance of these proteins for T2D or T2D-related traits. Among these three proteins, there was, however, no evidence of any drug development for ENTR1 and PON3.

Previous observational studies of proteomics and T2D have involved primarily European ancestry populations, used different study designs, and included varying number of proteins measured by different assay platforms.^13, 15, 16, 20^ Although there were inconsistent findings, several proteins have been consistently associated with T2D, including IGFBP1, IGFBP2, GHR and SHBG.^12, 13^ In the present study, observational analyses found that IGFBP1 and IGFBP2 were most strongly associated with T2D. The IGFBPs, which comprise some 15 proteins so far identified, importantly impact on systemic IGF signalling by modulating activity and decay of their binding partners. IGFBP-2, which is mainly released by the liver, directly supports glucose homeostasis by stimulating glucose uptake into adipocytes, and also inhibits adipo-genesis and enhances long-term insulin sensitivity.^28^ Consistent with previous findings, GHR was positively associated with T2D risk in the present study. There is evidence that high levels of GHR can accelerate systemic insulin resistance,^29^ which may partly explain the observed associations. Several previous studies reported possible protective associations between T2D risk and SHBG, which is a hepatokine that binds to circulating steroid hormones (testosterone, oestradiol) and acts on macrophages and adipocytes to suppress inflammation and lipid accumulation.^30^ Although the associations of SHBG with T2D became borderline significant after multiple testing correction (HR per SD higher: 0.69 [0.56-0.85]; 5% FDR *P*=0.057), the present results were consistent with previous study findings. Indeed, in CKB observational analyses, levels of adiponectin were inversely associated with risk of diabetes before multiple testing correction, consistent with previous literature.^31^ In genetic analyses, however, we did not found the causal roles of these few known proteins in aetiology of T2D, possibly due to limited study power. The present study also found significant protective associations of pancreatic alpha-amylase (AMY2A, AMY2B) with T2D. Amylase is a digestive enzyme predominantly secreted by the pancreas and salivary glands and acts as a catalyst for carbohydrate hydrolysis, which is one of the viable targets to control T2D. Despite consistent observational finings, few previous studies were able to assess the causal relevance of these proteins in T2D. In our study, we confirmed the causal relevance of AMY2B in UKB using different *cis*-pQTL (OR per SD higher: 0.94 [0.91-0.97]; *P*=0.0003). On the other hand, we found three proteins (PON3, LPL and ENTR1) were significantly associated with T2D in both observational and genetic analyses.

PON3 is expressed primarily in the liver and there is good evidence from animal experiment and epidemiologic studies that PON3 can inhibit oxidative stress, suppress inflammation, improve insulin resistance and abnormal glucolipid metabolism, and protect against atherosclerosis.^32^ As in the present study, the inverse associations between PON3 and incident T2D were also reported in two previous prospective studies in Sweden (1026 participants with 146 incident T2D cases) and Germany (1143 participants with 178 incident T2D cases),^16, 33^ which persisted after adjusting for plasma glucose (marginal significant in the present study), implying a glucose independent association with T2D incidence.^16^ However, no previous genetic studies supported its causal relevance for T2D incidence, therefore findings from the present study provides strong and novel support for PON3 as a potential target for improved prevention and treatment of T2D.

LPL (Lipoprotein lipase) is a rate-limiting enzyme that hydrolyzes circulating triglyceride-rich lipoproteins including very low-density lipoproteins and chylomicrons.^34^ This enzyme is predominantly located in adipose tissue, muscle and cardiac tissue, and a reduction in LPL activity is associated with an increase in plasma levels of triglycerides, prompting evaluation of target druggability for treatment of dyslipidemia,^34^ but its relevance to insulin resistance and glucose metabolism is less clear. Previous MR analyses suggested potential causal effects of LPL on insulin levels and the development of T2D.^33^ Moreover, a pharmacological study involving 392,220 Europeans showed that triglyceride-lowering alleles in the LPL were associated with lower risk of T2D, independent of LDL-C lowering genetic mechanisms.^35^ These findings provide genetic support for the development of agents that enhance LPL-mediated lipolysis for T2D prevention, which suggests, if further confirmed in other studies, potential opportunities for drug-repurposing for the treatment of T2D.

None of the previous observational studies have examined the associations of plasma levels of ENTR1 with risk of T2D. The *ENTR1* gene encodes the endosome associated trafficking regulator 1, which has a potential role in the transcriptional regulation of the solute carrier family 2 member 1 glucose 40 transporter protein (SLC2A1).^36^ Importantly, SLC2A1 is responsible for approximately 30−40% of the glucose uptake in skeletal muscle, with the remainder transported through GLUT4.^36^ This may partially explain the strong and apparently causal associations with T2D observed in the present study.

However, we could not exclude the possibility that these associations were caused by other pathways and further investigations of ENTR1 as a potential novel target for T2D are warranted.

In recent years, various proteomic-based prediction models for prevalent or incident diabetes have been developed, with varying number of proteins included (3 to 1468) and largely different degree of predictive performance.^9, 12, 14^ More recently, UKB developed a ProteinScore for T2D based on 1468 OLINK proteins, which outperformed a polygenic risk score and HbA1C.^9^ In the absence of HbA1c, we found that the addition of 33 or even 10 top proteins to conventional risk factors (including blood glucose) significantly improve the risk prediction of incident T2D in Chinese adults. Moreover, the same proteins identified in Chinese adults also yielded comparable results in European populations so could be considered for future clinical application in diverse populations.

The chief strengths of the present study include the large number of proteins assayed, independent replication of the main results internally and externally, use of ancestry-specific genetic instruments to assess causality, exclusion of CKB data from AGEN T2D GWAS summary statistics to minimize potential collider bias resulting from sample overlap, and multiple downstream analyses to assess possible mechanisms underlying these associations. Moreover, we also assessed the utility of proteomic-based risk prediction for T2D in diverse populations, independent and in combination with conventional risk factors. However, the present study also had several limitations. First, the study sample size was modest in CKB, limiting its power to detect more significant associations. Second, we were unable to independently replicate the observational findings in other East Asian populations due to the lack of available data. However, internal replication with prevalence T2D and plasma glucose levels, and external replication with incident T2D, glucose and HbA1c in UKB confirmed the validity of our observational findings when applying similar multiple test correction. Third, the two-sample MR analyses only involved two thirds of proteins due to lack of overlapping *cis*-pQTLs in publically-available GWAS summary statistics. Fourth, there was no kidney function data collected in CKB among the study participants. In UKB, however, further adjustment for kidney function (blood creatinine) had minor effect on the total number of proteins associated with incident diabetes (1514 with adjustment vs 1541 without adjustment, at FDR<0.05). Future studies with a larger sample size and better genetic instruments, involving perhaps both *cis*- and *trans*-pQTLs, more advanced method of machine learning in risk prediction, and functional analyses are needed to further identify, replicate and clarify the associations of different proteins with T2D in different ancestry populations.

In summary, the present study identified 33 proteins that were significantly associated with T2D, with strong genetic support for the causal relevance of three proteins. With the exception of one protein (LPL), there was no evidence of any drug development for two proteins, particularly PON3, which is highly expressed in liver cells and is a promising drug target for improved prevention and treatment of T2D. Further biological validation using *in vitro* and *in vivo* experiments along with human studies are required to elucidate the underlying mechanisms. The present study highlighted the importance of proteomics in prospective studies of diverse populations to improve risk prediction, enhance understanding of disease aetiology and discover potential novel drug targets for treatment and prevention of T2D as well as other diseases.

## Supplementary Material

Supplementary Material

## Figures and Tables

**Table 1:** Predictive values of conventional risk factors, RPG, and 33 proteins for incident T2D, separately and combined

Model	AUC	NRI (95% CI)
Base model ^a^	0.754 (0.710-0.798)	
Random plasma glucose (RPG)	0.646 (0.591-0.700)	
33 proteins	0.824 (0.786-0.862)	
		
Base model + RPG	0.774 (0.730-0.818)	14% (3-25%)^b^
Base model + 33 proteins	0.874 (0.844-0.904)	36% (22-50%)^b^
RPG + 33 proteins	0.829 (0.791-0.868)	43% (32-54%)^c^
Base model + RPG + 33 proteins	0.876 (0.846-0.906)	38% (24-52%)^d^
Base model + RPG + top 10 proteins^e^	0.844 (0.803-0.885)	28% (19-37%)^d^

a Predictors in the base model included age, sex, study area, fasting time, education, smoking, alcohol consumption, physical activity, family history of diabetes and BMI.

b Reference: Base model

c Reference: RPG

d Reference: Base model + RPG

e Ordered by *P* value

**Table 2:** Genetic effect estimates, colocalization, PheWAS results, and relevant drug targets of three proteins showing genetic effects on T2D

Protein	Full name	Two-sample MR			PH4	PheWAS associations	Drug (indication)
		*cis*-pQTL	OR (95% CI) per SD higher	*P-value*			
ENTR1	Endosome-associated-trafficking regulator 1	rs1051957	1.26 (1.18-1.34)	1.3E-11	0.01	T2D, HbA1c, glucose, insulin	─
LPL	Lipoprotein lipase	rs17411113	0.91 (0.85-0.97)	0.0098	0.87	T2D, MI, CAD, TG, VLDL, ApoA	Ibrolipim (lipid-lowering)
PON3	Serum paraoxonase/ lactonase 3	rs1053275	0.94 (0.89-0.98)	0.0047	0.65	ApoA, LDL	─

## Data Availability

The China Kadoorie Biobank (CKB) is a global resource for the investigation of lifestyle, environmental, blood biochemical and genetic factors as determinants of common diseases. The CKB study group is committed to making the cohort data available to the scientific community in China, the UK and worldwide to advance knowledge about the causes, prevention and treatment of disease. For detailed information on what data is currently available to open access users and how to apply for it, please visit: http://www.ckbiobank.org/site/Data+Access. A research proposal will be requested to ensure that any analysis is performed by *bona fide* researchers. Researchers who are interested in obtaining additional information or data that underlines this paper should contact ckbaccess@ndph.ox.ac.uk. For any data that are not currently available for open access, researchers may need to develop formal collaboration with study group.

## Article Highlights

- ***Why did we undertake this study?***
  Proteo-genomic analyses in prospective studies can help discover novel protein biomarkers for T2D.
- ***What is the specific question(s) we wanted to answer?***
  We assessed the associations of 2923 proteins with risk of incident T2D, utility of specific proteins for risk prediction and druggability of certain causal proteins.
- ***What did we find?***
  Thirty-three proteins were associated with incident T2D. Adding these 33 proteins to the conventional risk factors substantially improved T2D risk prediction. Genetic analyses identified causal relevance of three proteins, with one (PON3) highly expressed in liver tissue and potential relevant for T2D prevention and treatment.
- ***What are the implications of our findings?***
  These findings highlighted the importance of proteomics in prospective studies to improve risk prediction and discover novel drug targets for T2D.

## Twitter summary

Integrated proteomic and genetic analyses in diverse populations identified novel protein biomarkers for improved risk prediction and development of novel drug targets for type 2 diabetes

## Code availability

Custom code was used all statistical analyses in this report.

## Abbreviations

AAGEN: Asian Genetic Epidemiology Network
AUC: Area under the curve
CAD: Coronary artery disease
*cis*-pQTL: *cis* protein quantitative trait locus
CKB: China Kadoorie Biobank
CKB: Creatine kinase B-type
CPM: Carboxypeptidase M
CTSD: Cathepsin D
DIAMANTE: Diabetes Meta-Analysis of Trans-Ethnic association studies
ENTR1: Endosome-associated-trafficking regulator 1
ESM1: Endothelial cell-specific molecule 1
FDR: False discovery rate
FGFBP1: Fibroblast growth factor-binding protein 1
GHR: Growth hormone receptor
GLB1: Beta-galactosidase
GO: Gene Ontology
GTEx: Genotype-Tissue Expression
GWAS: Genome-wide association study
HbA1c: Hemoglobin A1c
HR: Hazards ratio
HuGE: Human Genetic Evidence
ICD-10: International Classification of Diseases 10th Revision
IHD: Ischemic heart disease
IGFBP: Insulin-like growth factor-binding protein
IGSF9: Protein turtle homolog A
IL18R1: Interleukin-18 receptor 1
KEGG: Kyoto Encyclopedia of Genes and Genomes
LOD: Limit of detection
LPL: Lipoprotein lipase
MI: Myocardial infarction
MR: Mendelian randomization
NPX: Normalized Protein eXpression
NRI: Net reclassification index
OR: Odds ratio
PheWAS: Phenome-wide association study
PON3: Serum paraoxonase/lactonase 3
PRDX2: Peroxiredoxin 2
PRCP: Lysosomal Pro-X carboxypeptidase
RIDA: 2-iminobutanoate/2-iminopropanoate deaminase
SD: Standard deviation
SHBG: Sex hormone-binding globulin
SLC2A1: Solute carrier family 2 member 1
T2D: Type 2 diabetes
T2DKP: Type 2 Diabetes Knowledge Portal
UKB: UK Biobank
VNN1: Pantetheinase
