# Supplementary material for: Proteomic analyses in diverse populations improved risk prediction and identified new drug targets for type 2 diabetes
Source: Diabetes Care. Author manuscript; Available in PMC 2024 Jun 1. (PMC7615965; doi:10.2337/dc23-2145)

Proteomic analyses in diverse populations improved risk prediction and identified new drug targets for type 2 diabetes

Supplementary Material

Contents

Members of the China Kadoorie Biobank collaborative group 2
eAppendix 3
eTable 1. Baseline characteristics of participants in proteomic subcohort and genotyped cohort in CKB 5
eTable 2. Continuous NRI of prediction models of conventional risk factors, RPG, and 33 proteins for incident T2D, separately and combined 6
eTable 3. Baseline characteristics of participants in UKB 7
eTable 4. Predictive values of conventional risk factors, RPG and proteins, separately and combined, for incident T2D in UKB 8
eFigure 1. Adjusted HRs for risk of diabetes by quartiles of 33 significant proteins 9
eFigure 2. Associations of 1-SD higher levels of 33 significant proteins with a) incident T2D, b) prevalent T2D and c) RPG, respectively 10
eFigure 3. Associations of 1-SD higher levels of 2941 proteins with a) prevalent diabetes, b) RPG levels and c) number of proteins overlapped with incident T2D in observational analyses 11
eFigure 4. Adjusted HRs for T2D associated with 1-SD higher levels of 33 significant proteins (OLINK batch 1) in a) CKB and b) UKB, respectively 12
eFigure 5. Correlation matrix of 33 proteins significantly associated with risk of incident T2D 13
eFigure 6. Calibration plot of risk prediction models for T2D 14
eFigure 7. Chord diagrams of enriched GO molecular functions for 33 proteins significantly associated with risk of T2D 15

CONFIDENTIAL-For Peer Review Only

Members of the China Kadoorie Biobank collaborative group

International Steering Committee: Junshi Chen, Zhengming Chen (PI), Robert Clarke, Rory Collins, Liming Li (PI), Chen Wang, Jun Lv, Richard Peto, Robin Walters.

International Co-ordinating Centre, Oxford: Daniel Avery, Derrick Bennett, Ruth Boxall, Sushila Burgess, Ka Hung Chan, Yiping Chen, Zhengming Chen, Johnathan Clarke, Robert Clarke, Huaidong Du, Ahmed Edris Mohamed, Hannah Fry, Simon Gilbert, Pek Kei Im, Andri Iona, Maria Kakkoura, Christiana Kartsonaki, Hubert Lam, Kuang Lin, James Liu, Mohsen Mazidi, Iona Millwood, Sam Morris, Qunhua Nie, Alfred Pozarickij, Paul Ryder, Saredo Said, Dan Schmidt, Becky Stevens, Iain Turnbull, Robin Walters, Baihan Wang, Lin Wang, Neil Wright, Ling Yang, Xiaoming Yang, Pang Yao.

National Co-ordinating Centre, Beijing: Xiao Han, Can Hou, Qingmei Xia, Chao Liu, Jun Lv, Pei Pei, Dianjanyi Sun, Canqing Yu,.

10 Regional Co-ordinating Centres:

Guangxi Provincial CDC: Naying Chen, Duo Liu, Zhenzhu Tang. Liuzhou CDC: Ningyu Chen, Qilian Jiang, Jian Lan, Mingqiang Li, Yun Liu, Fanwen Meng, Jinhuai Meng, Rong Pan, Yulu Qin, Ping Wang, Sisi Wang, Liuping Wei, Liyuan Zhou. **Gansu** Provincial CDC: Caixia Dong, Pengfei Ge, Xiaolan Ren. Maiji CDC: Zhongxiao Li, Enke Mao, Tao Wang, Hui Zhang, Xi Zhang. **Hainan** Provincial CDC: Jinyan Chen, Ximin Hu, Xiaohuan Wang. Meilan CDC: Zhendong Guo, Huimei Li, Yilei Li, Min Weng, Shukuan Wu. **Heilongjiang** Provincial CDC: Shichun Yan, Mingyuan Zou, Xue Zhou. Nangang CDC: Ziyan Guo, Quan Kang, Yanjie Li, Bo Yu, Qinai Xu. **Henan** Provincial CDC: Liang Chang, Lei Fan, Shixian Feng, Ding Zhang, Gang Zhou. Huixian CDC: Yulian Gao, Tianyou He, Pan He, Chen Hu, Huarong Sun, Xukui Zhang. **Hunan** Provincial CDC: Biyun Chen, Zhongxi Fu, Yuelong Huang, Huilin Liu, Qiaohua Xu, Li Yin. Liuyang CDC: Huajun Long, Xin Xu, Hao Zhang, Libo Zhang. **Jiangsu** Provincial CDC: Jian Su, Ran Tao, Ming Wu, Jie Yang, Jinyi Zhou, Yonglin Zhou. Suzhou CDC: Yihe Hu, Yujie Hua, Jianrong Jin, Fang Liu, Jingchao Liu, Yan Lu, Liangcai Ma, Aiyu Tang, Jun Zhang. **Qingdao** CDC: Liang Cheng, Ranran Du, Ruqin Gao, Feifei Li, Shanpeng Li, Yongmei Liu, Feng Ning, Zengchang Pang, Xiaohui Sun, Xiaocao Tian, Shaojie Wang, Yaoming Zhai, Hua Zhang, Licang CDC: Wei Hou, Silu Lv, Junzheng Wang. **Sichuan** Provincial CDC: Xiaofang Chen, Xianping Wu, Ningmei Zhang, Weiwei Zhou. Pengzhou CDC: Xiaofang Chen, Jianguo Li, Jiaqiu Liu, Guojin Luo, Qiang Sun, Xunfu Zhong. **Zhejiang** Provincial CDC: Weiwei Gong, Ruying Hu, Hao Wang,Meng Wang, Min Yu. Tongxiang CDC: Lingli Chen, Qijun Gu, Dongxia Pan, Chunmei Wang, Kaixu Xie, Xiaoyi Zhang.

eAppendix

Covariates selection in CKB

In our exploratory analyses, ambient temperature (temperature recorded on the date of blood collection) was associated with levels of ~40% proteins. Given the substantial variation of ambient temperature among 10 study areas (60℃ difference: from -25℃ to 35℃) in CKB, it is important to consider it as a covariate in the model.

The UK Biobank sample population

UK Biobank (UKB) is a population-based cohort of around 500,000 individuals aged between 40-69 years that were recruited between 2006 and 2010. Genome-wide genotyping, exome sequencing, electronic health record linkage, whole-body magnetic resonance imaging, blood and urine biomarkers and physical and anthropometric measurements are available. More information regarding the full measurements can be found at: https://biobank.ndph.ox.ac.uk/showcase/. The UK Biobank Pharma Proteomics Project (UKB-PPP) is a precompetitive consortium of 13 biopharmaceutical companies funding the generation of blood-based proteomic data from UKB volunteer samples (**eTable 3**).

Proteomics in the UK Biobank

The UKB-PPP sample includes 54,306 UKB participants and 2923 unique proteins measured using The Olink technology uses Proximity Extension Assay. A randomised subset of 46,673 individuals were selected from baseline UKB, with 6,385 individuals selected by the UKB-PPP consortium members and1,268 individuals included that participated in a COVID-19 study. The randomised samples have been shown to be highly representative of the wider UKB population and they are used in the current analyses, whereas the consortium selected individuals were enriched for 122 diseases. Details on sample selection for UKB-PPP, in addition to processing and quality control information for the Olink assay are provided in elsewhere (Nature 2023; *DOI: 10.1038/s41586-023-06592-6*).

Electronic health data linkage in the UK Biobank

Electronic health linkage to NHS records was used to collate incident diagnoses. Death information was sourced from the death registry data available through the UK Biobank.

CONFIDENTIAL-For Peer Review Only

Cancer outcomes were sourced from the cancer registry (ICD codes), whereas non-cancer diseases were sourced from first occurrence traits available in the UK Biobank. The first occurrence traits integrate GP (read2/3), ICD (9/10) with self-report and ICD codes present on the death registry to identify the earliest date of diagnoses. These data sources are linked to 3-digit ICD trait codes.

Statistical analyses

Plasma protein levels were standardized (i.e. values of each protein were divided by their SD) and analysed as continuous variables. In observational analysis, Cox regression models were used to estimate adjusted HRs (and 95% CI) for incident. All analyses were adjusted for age, age^2, sex, assess center, fasting time, education, smoking status, alcohol intake frequency, physical activity and BMI. All analyses were restricted to participants without prior diabetes at baseline. All statistical analyses were performed using R version 4.1.2. Benjamini-Hochberg FDR was used to correct for multiple testing.

CONFIDENTIAL-For Peer Review Only

eTable 1. Baseline characteristics of participants in proteomic subcohort and genotyped cohort in CKB

Characteristics [a]	Proteomic subcohort (n=1896)	Genotyped CKB (n=79,159)
**Demographic and lifestyle factors**		
Age, years, mean (SD)	51.3 (10.4)	51.7 (10.6)
Women, %	62.1	60.2
Urban residents, %	50.6	45.4
Education ≥high school, %	21.4	21.5
Ever regular smoker in men, %	74.9	74.0
Ever regular smoker in women, %	3.3	3.1
Ever regular drinker in men, %	40.1	36.9
Ever regular drinker in women, %	2.7	2.4
Physical activity, MET-h/day, mean (SD)	21.3 (12.3)	21.3 (13.9)
**Medical history and health status, %**		
Self-rated poor health	13.4	10.2
Chronic kidney disease	1.4	1.3
Cancer	0.6	0.5
Family history of diabetes [b]	16.1	15.8
**Anthropometry and blood pressure, mean (SD)**		
BMI, kg/m^2	23.9 (3.2)	23.6 (3.4)
WC, cm	80.3 (8.7)	80.1 (9.7)
SBP, mmHg	131 (20)	130 (21)
RPG, mmol/L	6.1 (2.5)	6.0 (2.3)
Fasting time, hours	5.1 (4.3)	5.2 (4.2)

[a] Adjusted for age, sex and study area, as appropriate.
[b] Family history of diabetes represents diabetes history of any first-degree relative
Abbreviations: SD=Standard deviation; BMI=Body mass index; SBP=Systolic blood pressure; MET=Metabolic equivalent of task; RPG=Random plasma glucose; WC=Waist circumference

CONFIDENTIAL-For Peer Review Only

eTable 2. Continuous NRI of prediction models of conventional risk factors, RPG, and 33 proteins for incident T2D, separately and combined

Model	NRI (95% CI)
Base model [a]	
Random plasma glucose (RPG)	
33 proteins	
Base model + RPG	44% (23-65%)[b]
Base model + 33 proteins	106% (88-124%)[b]
RPG + 33 proteins	98% (80-116%)[c]
Base model + RPG + 33 proteins	97% (79-115%)[d]
Base model + RPG + top 10 proteins [e]	84% (65-103%)[d]

a Predictors in the base model included age, sex, study area, fasting time, education, smoking, alcohol consumption, physical activity, family history of diabetes and BMI.
b Reference: Base model
c Reference: RPG
d Reference: Base model + RPG
e Ordered by P value

CONFIDENTIAL-For Peer Review Only

eTable 3. Baseline characteristics of participants in UKB

**Characteristics**	**UKB-PPP Randomized baseline** (n=46595)	**UKB full cohort**
Demographic and lifestyle factors		
Age, years, mean (SD)	56.7 (8.1)	56.5 (8.1)
Women, %	54.3	54.4
Townsend Deprivation Index	-1.23 (3.15)	-1.29 (3.09)
Smoking		
Never	54.3%	54.8%
Previous	34.8%	34.6%
Current	10.9%	10.6%
Ethnic background		
Asian/Asian British	2.0%	2.0%
Black/Black British	1.7%	1.6%
Chinese	0.3%	0.3%
Mixed	0.7%	0.6%
White	94.4%	94.6%
Other ethnic group	1.0%	0.9%
ABO blood group		
O	43.2%	43.3%
A	43.4%	43.5%
B	9.7%	9.6%
AB	3.7%	3.6%
Anthropometry, mean (SD)		
BMI, kg/m^2	27.4 (4.8)	27.4 (4.8)
Biochemistry		
Alanine aminotransferase (U/l)	23.41 (14.28)	23.55 (14.18)
Alkaline phosphatase (U/l)	83.74 (26.3)	83.67 (26.46)
Aspartate aminotransferase (U/l)	26.27 (11.09)	26.23 (10.66)
Cholesterol (mmol/l)	5.69 (1.15)	5.69 (1.14)
Creatinine (umol/l)	72.28 (18.91)	72.31 (18.55)
Gamma glutamyltransferase (U/l)	37.29 (41.11)	37.39 (42.09)
Glucose (mmol/l)	5.13 (1.25)	5.12 (1.24)
Glycated haemoglobin (HbA1c) (mmol/mol)	36.20 (6.86)	36.13 (6.78)
HDL-cholesterol (mmol/l)	1.45 (0.38)	1.45 (0.38)
Triglycerides (mmol/l)	1.75 (1.03)	1.75 (1.03)

CONFIDENTIAL-For Peer Review Only

eTable 4. Predictive values of conventional risk factors, RPG and proteins, separately and combined, for incident T2D in UKB

Model	AUC (95% CI)
Base model [a]	0.796 (0.787-0.805)
Random plasma glucose (RPG)	0.738 (0.726-0.749)
33 proteins	0.890 (0.840-0.857)
Base model + RPG	0.852 (0.843-0.861)
Base model + 33 proteins	0.879 (0.870-0.887)
RPG + 33 proteins	0.882 (0.873-0.891)
Base model + RPG + 33 proteins	0.893 (0.884-0.906)
HbA1c	0.892 (0.884-0.899)
Base model + RPG + HbA1c	0.917 (0.910-0.923)
RPG + HbA1c + 33 proteins	0.931 (0.924-0.938)
Base model + RPG + HbA1c + 33 proteins	0.937 (0.930-0.944)

[a] Adjusted for age, sex, study area, fasting time, education, smoking, alcohol consumption, physical activity, family history of diabetes and BMI.

eFigure 1. Adjusted HRs for risk of diabetes by quartiles of 33 significant proteins

The sizes of the data markers are proportional to the inverse of the variance of the log HRs. The numbers above the 95% CI are point estimates for HRs, and the numbers below are numbers of diabetes cases for each category. The models were adjusted for sex, age, age², region, fasting time, ambient temperature, plate ID, education, smoking, alcohol consumption, physical activity, family history of diabetes and BMI.

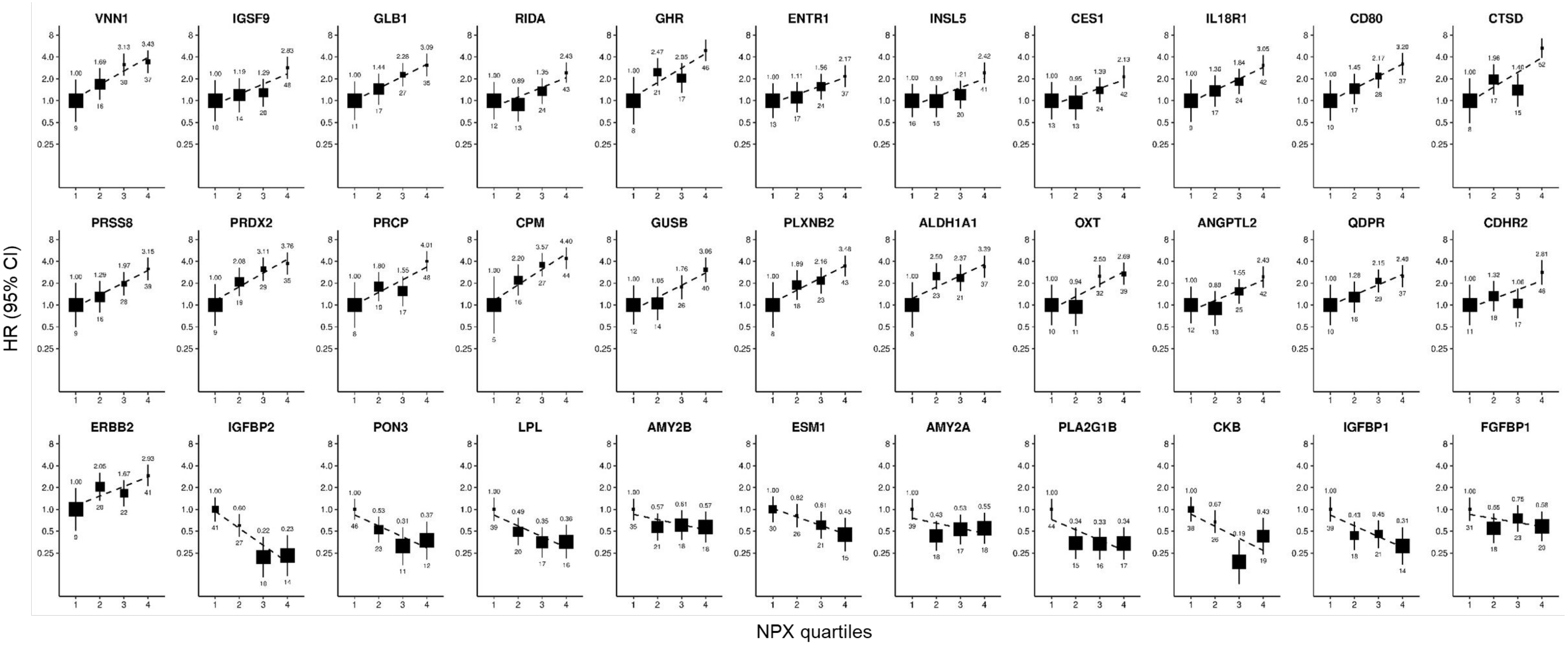

eFigure 2. Associations of 1-SD higher levels of 33 significant proteins with a) incident T2D, b) prevalent T2D and c) RPG, respectively

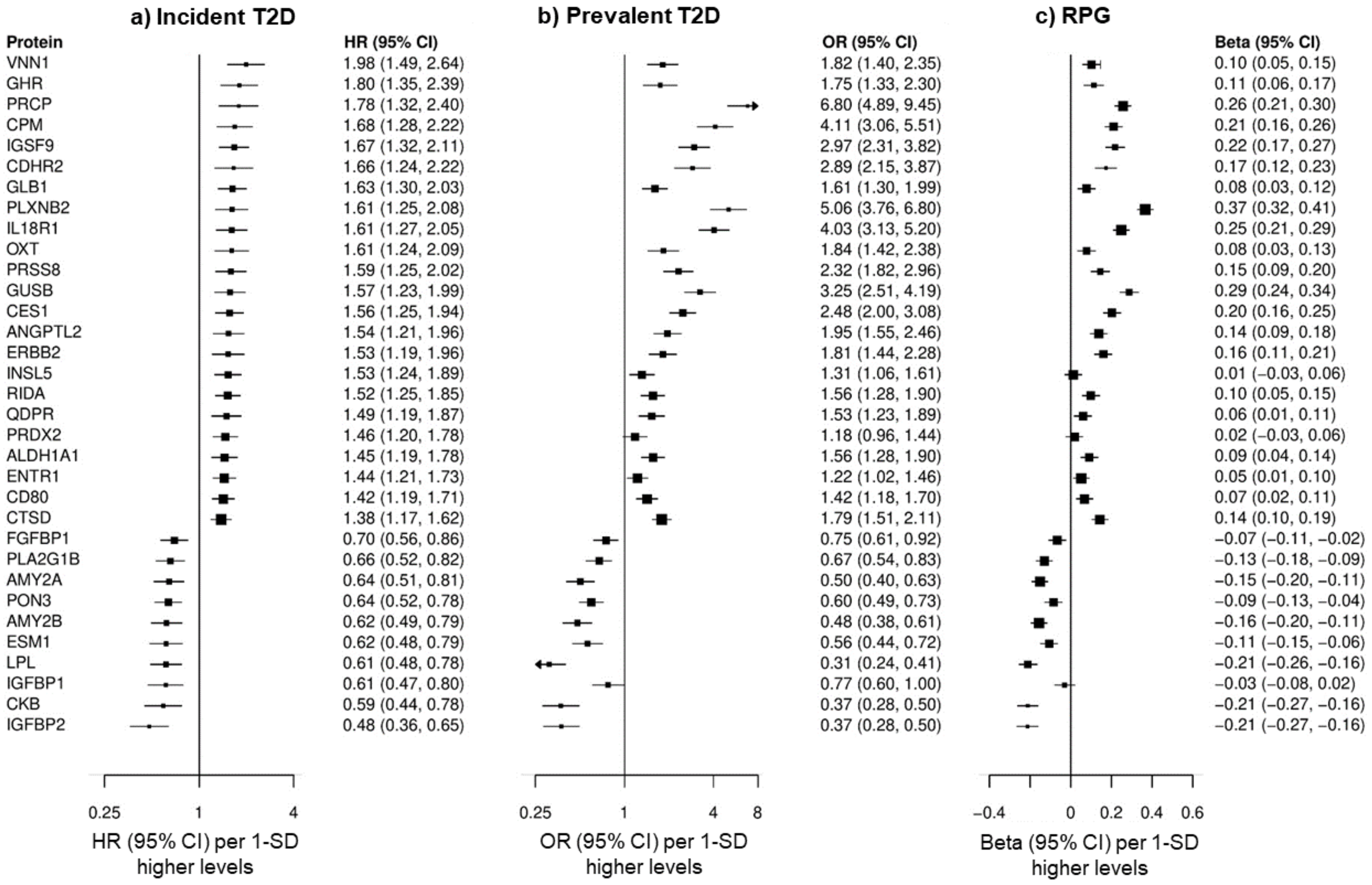

eFigure 3. Associations of 1-SD higher levels of 2941 proteins with a) prevalent diabetes, b) RPG levels and c) number of proteins overlapped with incident T2D in observational analyses

Models were adjusted for age, age square, sex, study area, fasting time, ambient temperature, plate ID, education, smoking, alcohol consumption, physical activity, family history of diabetes and BMI. Red, blue and grey dots denote positive significant, inverse significant and non-significant associations, respectively.

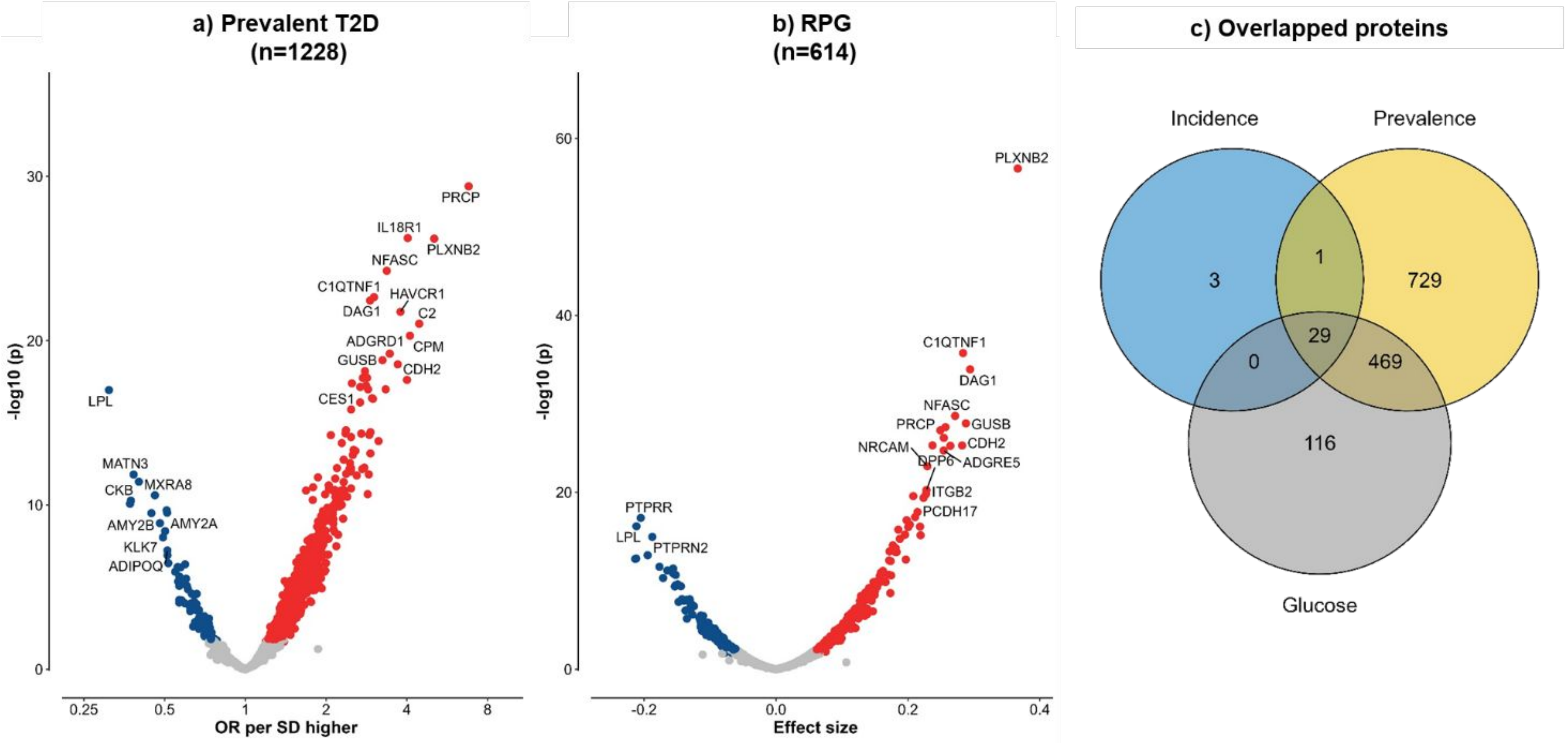

CONFIDENTIAL-For Peer Review Only

eFigure 4. Adjusted HRs for T2D associated with 1-SD higher levels of 33 significant proteins (OLINK batch 1) in a) CKB and b) UKB, respectively

Models were adjusted for age, age^2, sex, study area, fasting time, ambient temperature (CKB only), plate ID, education, smoking, alcohol consumption, physical activity, family history of diabetes and BMI. The comparison was conducted among 33 proteins from OLINK 2 batches.

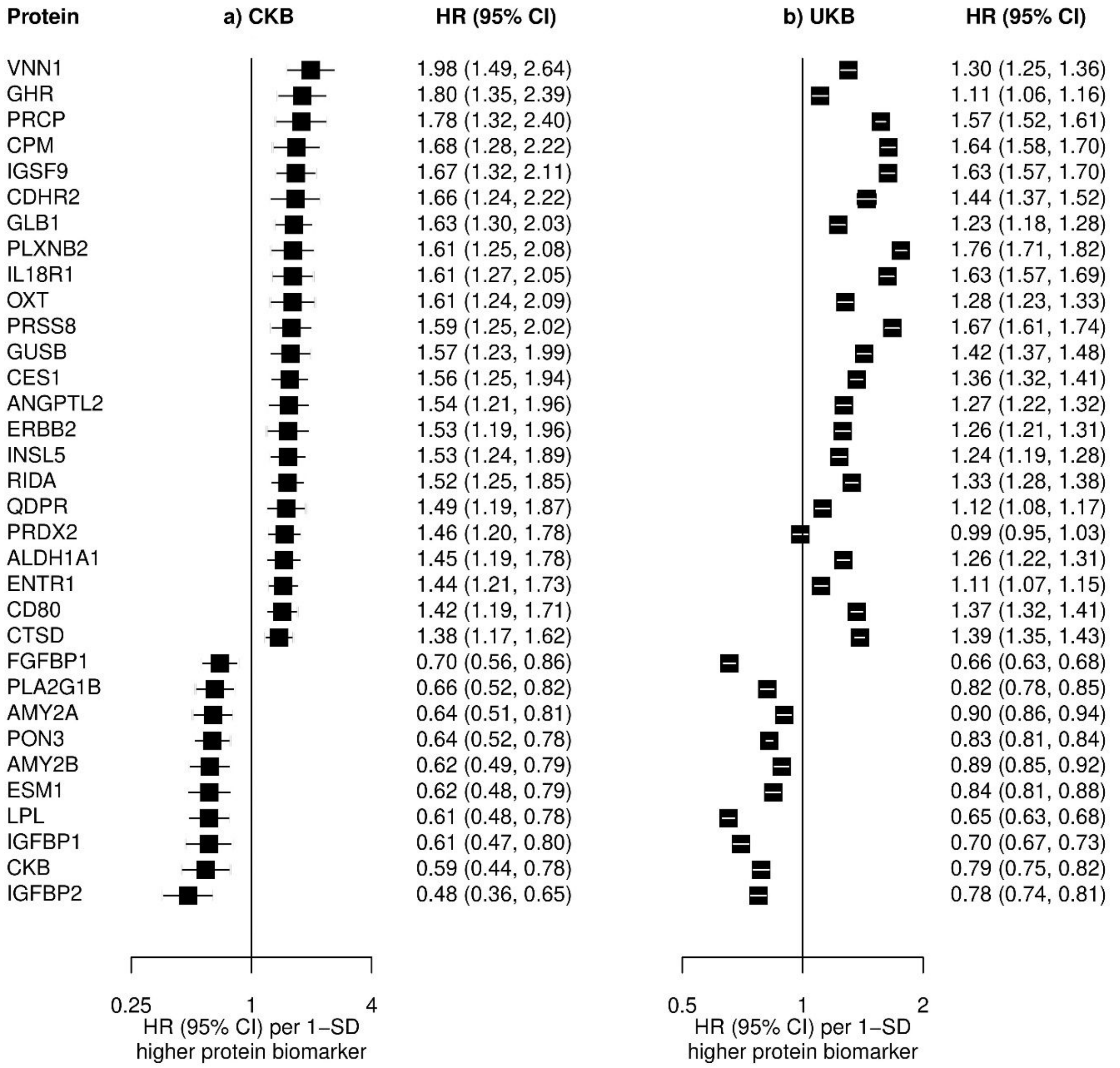

eFigure 5. Correlation matrix of 33 proteins significantly associated with risk of incident T2D

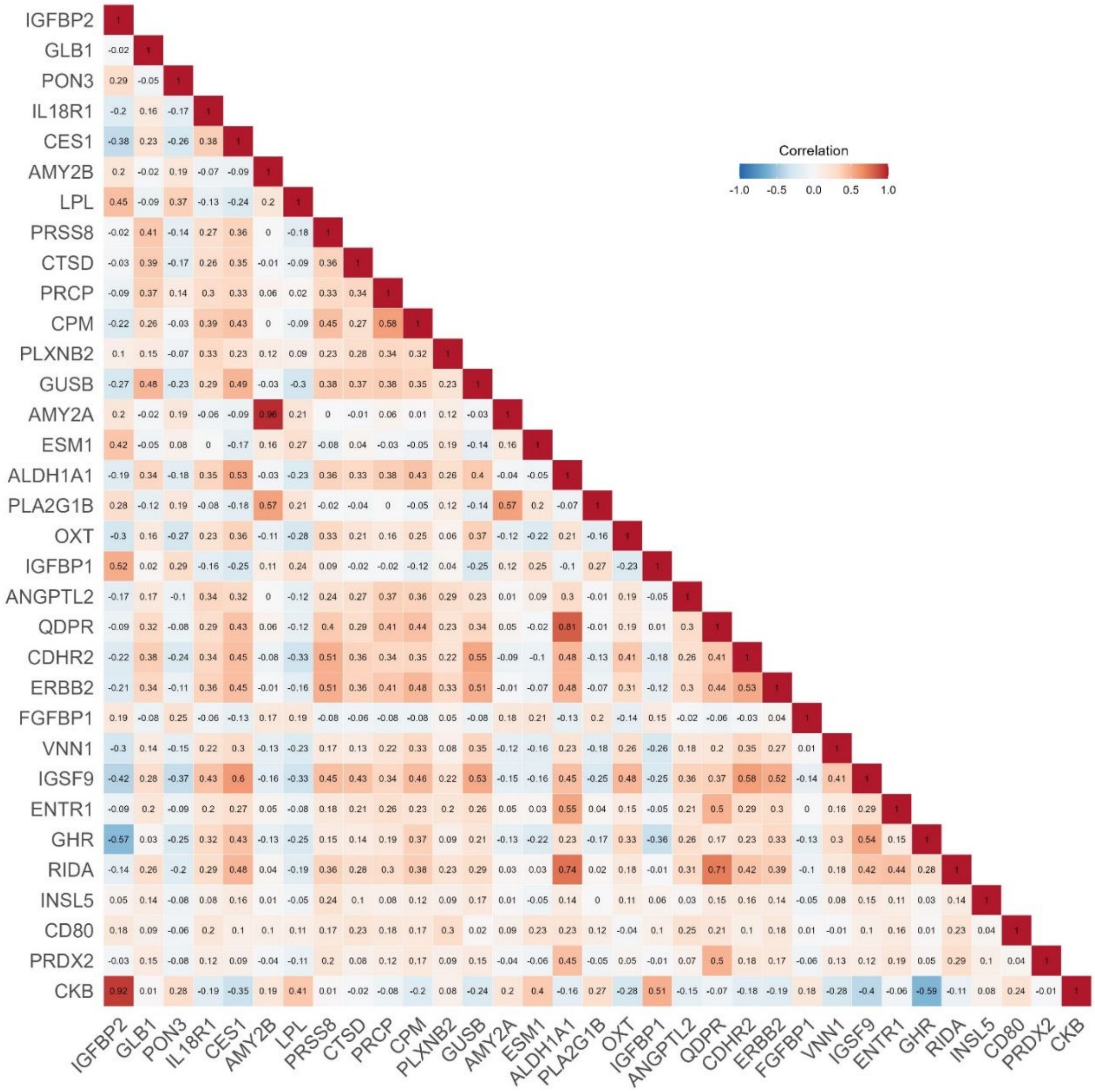

CONFIDENTIAL-For Peer Review Only

eFigure 6. Calibration plot of risk prediction models for T2D

The predictors in the model included age, sex, study area, fasting time, education, smoking, alcohol consumption, physical activity, family history of diabetes, BMI, RPG, and 33 proteins

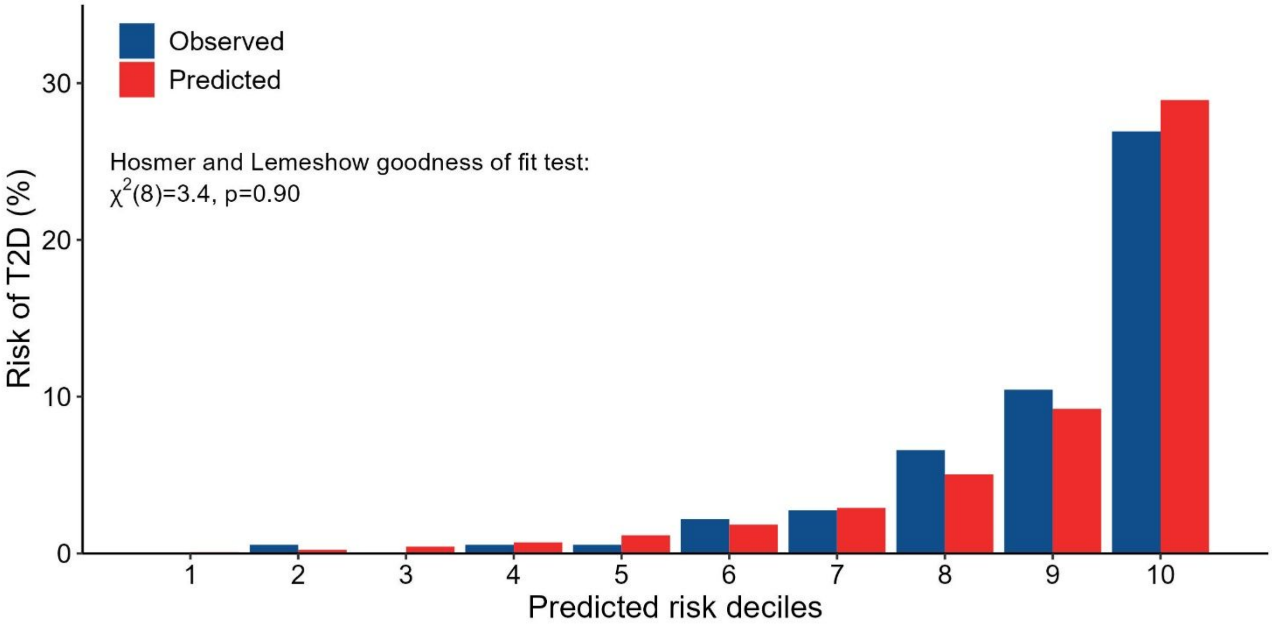

CONFIDENTIAL-For Peer Review Only

eFigure 7. Chord diagrams of enriched GO molecular functions for 33 proteins significantly associated with risk of T2D

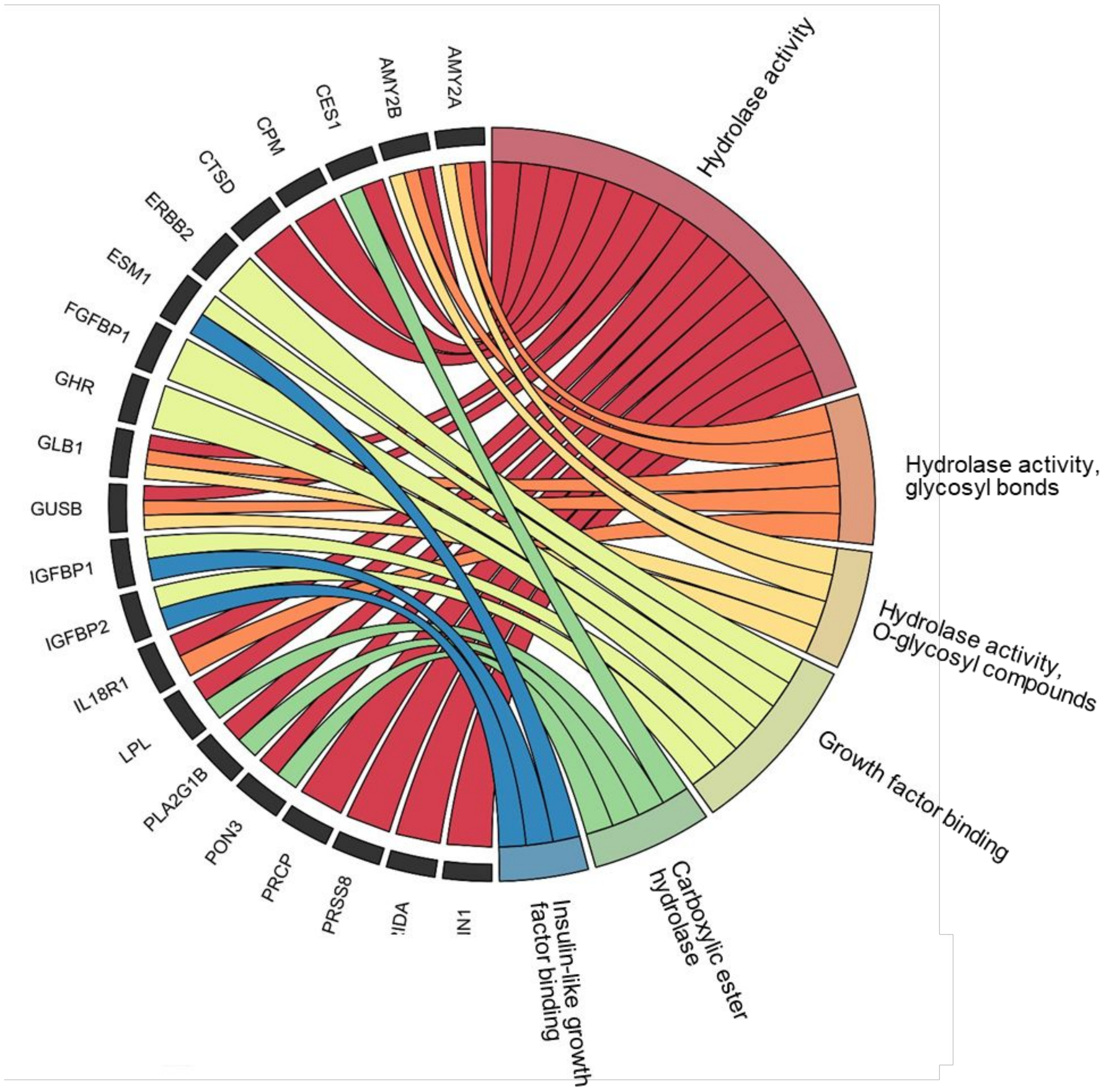

Supplement: Supplementary Material [file EMS195526-supplement-Supplementary_Material.pdf]
